# Factors Associated With Systemic Corticosteroid Use in Patients With Asthma: A Real-Life Study

**DOI:** 10.7759/cureus.97565

**Published:** 2025-11-23

**Authors:** Gregorio Alvarez-Vera, Lourdes Avila, Elsy M. Navarrete-Rodriguez, Joaquin A Pimentel-Hayashi, Carlos Macouzet-Sanchez, Edith Vallejo-Perez, Eduardo Pineyro-Beltran, Roxana Rodriguez-Romo, Pablo Contreras

**Affiliations:** 1 Allergy and Immunology, IMSS Hospital General de Zona No. 30, Iztacalco, Mexico City, MEX; 2 Allergy and Immunology, Hospital Infantil de Mexico Federico Gomez, Mexico City, MEX; 3 Allergy and Immunology, Respiratory and Immunology AstraZeneca, Mexico City, MEX; 4 Allergy and Immunology, Centro Regional de Alergia e Inmunología Clínica, Universidad Autonoma de Nuevo Leon, Hospital Universitario Dr. Jose Eleuterio Gonzalez, Monterrey, MEX; 5 Allergy and Immunology, ISSSTE Hospital General Vasco de Quiroga, Mexico City, MEX; 6 Allergy and Immunology, ISSSTE San Luis Potosi, Mexico City, MEX; 7 General Practice, Facultad de Medicina, Universidad Nacional Autonoma de Mexico, Mexico City, MEX; 8 General Practice, Facultad Mexicana de Medicina, Universidad La Salle, Mexico City, MEX

**Keywords:** allergic asthma, eosinophilic asthma, new-onset asthma, severe eosinophilic asthma, uncontrolled asthma

## Abstract

Introduction

Asthma is a chronic inflammatory respiratory disease that affects a substantial portion of the global population. Systemic corticosteroids (SCS) are generally reserved as a last-line therapy for severe asthma or acute exacerbations; however, their extended use is associated with serious adverse effects, including metabolic and musculoskeletal complications. In Mexico, documentation on SCS use among individuals with asthma is limited, highlighting the need to identify factors linked to their overuse to reduce long-term harm.

Materials and methods

A multicenter ambispective study was conducted in secondary and tertiary care hospitals in Mexico between June 2023 and June 2024. A total of 152 asthma patients were included, and clinical and laboratory data related to SCS use were collected. Variables analyzed included asthma severity, endotype, comorbidities, and the number of exacerbations in the past 12 months. Data were collected using standardized questionnaires and analyzed using descriptive and inferential statistics.

Results

A total of 152 patients were included in the final sample; 74 (48.7%) of the patients used SCS in the past 12 months. SCS use was more frequent in patients with severe asthma (81.8%) and eosinophilic endotype (83.3%), and these differences were statistically significant (p<0.05). Patients with a recent asthma diagnosis (<1 year) also exhibited high SCS use (90%), although this was not significantly different from those diagnosed for more than five years. Among comorbidities, all patients with nasal polyposis used SCS (p<0.05), whereas passive smoking and obesity showed no significant associations with SCS use. The median eosinophil count was higher in SCS users (208 vs. 114; p<0.05), reinforcing the relationship between eosinophilic inflammation and increased SCS use.

Conclusions

This study identifies factors associated with SCS use in asthma patients, including disease severity, eosinophilic inflammation, and nasal polyposis. These findings highlight the need for more specific therapeutic strategies, such as biologics, to reduce SCS dependency and associated adverse events. Despite certain limitations, the results highlight the importance of early diagnosis and effective asthma management to optimize treatment outcomes and minimize long-term risks.

## Introduction

Asthma is a heterogeneous, chronic, and inflammatory disease characterized by a history of respiratory symptoms such as wheezing, dyspnea, chest tightness, and coughing, which vary over time in frequency and intensity [1]. Asthma affects approximately 339 million people worldwide, and this number is projected to rise by an additional 100 million cases by 2030 [2]. The global prevalence of asthma symptoms is estimated to be 10% in children and adolescents and 6-7% in adults [3]. In Mexico, the prevalence of physician-diagnosed asthma in adults is 3.3% in men and 6.2% in women [4]. 

Between 25-40% of oral corticosteroid (OCS) prescriptions are attributable to respiratory diseases, particularly those associated with the airway [5]. For asthma specifically, the Global Initiative for Asthma (GINA) recommends OCS as a last-resort add-on maintenance therapy for patients at treatment step 5 who continue to have poor symptom control and/or frequent exacerbations despite good adherence and proper inhaler technique [1]. This guidance applies only after other contributing factors have been ruled out and alternative treatments, including biologics, have been considered. OCS use is also advised for the management of asthma exacerbations [1]. Based on these recommendations, OCS therapy should be limited to approximately 10% of patients with severe asthma and 10% of those experiencing an exacerbation at any given time.

A systematic review conducted by Bleecker et al. reported that in many cases, >10% of patients receive OCS treatment [6]. Accumulated OCS exposure is associated with an increased risk of developing acute and chronic adverse events related to the use of these medications [7], with the most common being osteoporosis, cardiovascular disease, and metabolic complications, including type 2 diabetes (hazard ratio (HR): 1.26) and obesity (HR: 1.14) [8]. Despite existing guideline recommendations, real-world evidence indicates that systemic corticosteroids (SCS) continue to be overprescribed.

The incidence of adverse events increases with each year of exposure, particularly when four or more OCS prescriptions are indicated per year. Sullivan et al. demonstrated that this is significantly associated with a higher likelihood of developing the following adverse events: osteoporosis (odds ratio (OR): 1.442; 95% confidence interval (CI): 1.278-1.626), hypertension (OR: 1.324; 95% CI: 1.202-1.458), obesity (OR: 1.280; 95% CI: 1.130-1.449), type 2 diabetes (OR: 1.299; 95% CI: 1.129-1.495), ulcers and/or gastrointestinal bleeding (OR: 1.330; 95% CI: 1.150-1.538), fractures (OR: 1.208; 95% CI: 1.042-1.401), and cataracts (OR: 1.256; 95% CI: 1.035-1.523) [9]. Regarding mortality, a study conducted in Sweden reported that prolonged OCS use was associated with an increased risk of all-cause mortality (adjusted HR: 1.34; 95% CI: 1.24-1.45; p<0.001) compared to periodic or non-use of these medications [10]. 

Over the years, several studies have sought to identify the characteristics of asthma patients who use oral corticosteroids or are at risk of requiring them as part of their treatment. A five-year retrospective cohort study in the United States reported that users of "high" doses of OCS were more likely to be older adults, women, have a history of severe asthma, a history of exacerbations, and a higher Charlson Comorbidity Index [11]. In a cross-sectional study in Portugal, four variables were found to be significantly associated with a higher likelihood of using OCS as part of asthma treatment: age ≤65 years, conjunctivitis, osteoporosis, and a history of at least one emergency health resource use in the previous 12 months [12]. Finally, in Latin America, Maspero et al. mentioned that the factors influencing the use of SCS in the region are diverse, highlighting the following: the prevalence of severe and/or uncontrolled asthma; the effectiveness, low cost, and availability of corticosteroids; and the experience, familiarity, or lack of knowledge regarding the use of these medications by treating physicians [13]. In Mexico, the SABINA-III cohort reported that 39.6% of the recruited patients had received at least one short course of SCS in the previous 12 months [14].

At present, there is limited evidence in our country on the use of OCS among asthma patients, underscoring the need to identify factors that may contribute to their overuse in order to reduce long-term adverse effects.

## Materials and methods

A retrospective and prospective multicenter study was conducted to collect data from asthma patients who were prescribed SCS in secondary- and tertiary-care hospitals. The data collection was primarily retrospective from medical records; however, for some patients, specific laboratory parameters such as peripheral blood eosinophil counts were obtained prospectively during clinical evaluation. Data collection spanned 12 months, from June 2023 to June 2024. A convenience sample of patients with mild, moderate, and severe asthma was included; these were patients seen by investigators in emergency departments or during first-time or follow-up consultations.

Inclusion criteria were patients aged six years and older with an asthma diagnosis confirmed via spirometry, medical history, or response to inhaled corticosteroid (ICS) treatment. Exclusion criteria included patients receiving SCS for conditions other than asthma (e.g., autoimmune diseases), those with a concomitant diagnosis of chronic obstructive pulmonary disease (COPD), or any other chronic respiratory disorder that could confound the analysis. Asthma severity was defined according to GINA recommendations based on the level of treatment required to achieve control: mild asthma corresponded to steps 1-2 (patients controlled with low-dose ICS or as-needed ICS-formoterol), moderate asthma to step 3 (requiring low-dose ICS-LABA maintenance therapy), and severe asthma to steps 4-5 (requiring medium- or high-dose ICS-LABA or additional controller therapy).

Clinical and laboratory variables associated with higher SCS use were analyzed. These variables included the severity of asthma based on ICS dosage as proposed by GINA 2023 [1], asthma endotype and phenotype, age, years since asthma diagnosis, sex, family history of asthma, the month in which the patient experienced exacerbations, the number of exacerbations in the past 12 months, prior hospitalizations due to asthma, ICU admissions due to asthma, allergic and non-allergic comorbidities, duration of continuous ICS use, and the number of salbutamol canisters used in the last 12 months. These patients were compared with those who had not used SCS in the last 12 months; physicians selected these patients presenting to outpatient clinics or the emergency department. Asthma phenotypes were defined based on clinical presentation (allergic, eosinophilic, adult-onset asthma), while endotypes were classified according to underlying inflammatory patterns, including T2-high (allergic or eosinophilic) and T2-low, as per the current GINA 2023 guidelines. 

Asthma severity was defined according to the GINA 2023 classification. Asthma endotypes were defined based on GINA 2023 criteria and routinely used biomarkers (IgE and blood eosinophil counts) [1]. All biomarkers were interpreted considering prior corticosteroid exposure. An allergic phenotype was identified with positive prick-test results or elevated IgE levels. The eosinophilic endotype was defined by an eosinophil count >150 cells/μL, the mixed phenotype included both allergic and eosinophilic endotypes, and the paucigranulocytic phenotype or low T2 inflammation was identified by the absence of type 2 inflammation biomarkers. These biomarkers were adjusted based on the lowest ICS dose the patient had used to account for modifications caused by SCS use. The number of years since diagnosis was obtained directly from the patient or the medical records. Asthma exacerbations were defined according to GINA 2023 as an increase or worsening of asthma symptoms and pulmonary function. The number of exacerbations was obtained from the patient's medical record. A short course of OCS was defined as OCS use for at least three days at a dose of 1 mg/kg/day [1].

To gather information on SCS use, patients were directly asked about their use of different types of SCS available in the country, including generic or brand names. Exclusion criteria included patients using SCS for any condition other than asthma. Data collection was centralized using standardized electronic forms. A total of five hospitals participated: three secondary-care hospitals and two tertiary-care hospitals located in three states of the country. The questions were prepared using appropriate medical terminology and avoiding ambiguous wording. Investigators reviewed the form before its use, and any doubts about the questions were clarified. In this study, the term SCS is used interchangeably to include intravenous, intramuscular, and oral corticosteroids, since all three routes of administration were analyzed.

Statistical analysis

Descriptive statistics were used to summarize demographic and clinical characteristics. Continuous variables were expressed as means and standard deviations (SD) or medians and interquartile ranges (IQR), depending on data distribution. Categorical variables were expressed as absolute and relative frequencies. Comparisons between patients who used SCS and those who did not were performed using the chi-square test (or Fisher’s exact test when appropriate) for categorical variables, and the Student’s t-test or Mann-Whitney U test for continuous variables. A p-value <0.05 was considered statistically significant. Statistical analyses were performed using STATA version 14. Given the exploratory, real-world nature of the study, no multivariable analyses were performed; instead, the focus was on identifying statistically and clinically relevant associations to guide future hypothesis-driven research.

Patients signed informed consent forms at the time of data collection. This study was approved by the Ethics and Research Committee of the Hospital Infantil de Mexico Federico Gomez (HIM-2023-012). This study followed the principles of the Declaration of Helsinki. No licensed or copyrighted assessment tools or scoring systems were used in this study. All definitions were based on the GINA 2023 guidelines, which are freely available for non-commercial research use [1].

## Results

A total of 152 patients were included in the final sample. The mean age of the patients was 42.9 years (SD 1.75). The sample consisted of 113 women (74.3%) and 39 men (25.7%), with 79% of patients recruited from secondary-care hospitals. Spirometry confirmed the asthma diagnosis in 75.2% of patients, while 24.8% of patients had a medical history suggestive of asthma symptoms along with a response to ICS treatment. Of the 152 patients, 74 (48.7%) used SCS in the last 12 months. Among those who used SCS, 17 patients (11.1%) had at least one short course of OCS in the last 12 months, and 29 patients (19%) had more than three short courses of OCS in the past year.

Regarding the duration of asthma diagnosis, 92 (60.5%) of patients had been diagnosed for more than five years, 49 (32.5%) between one and five years, and 10 (7%) for less than a year. Of those diagnosed with asthma for less than a year, nine (90%) used SCS, compared to 43 (48.4%) of patients with a diagnosis of more than five years. Only 10.2% of patients were first-time consultation cases. When comparing patients with less than one year of diagnosis to those with more than five years, the difference was not statistically significant. In terms of family history of asthma, 48.7% of patients reported no family history, while 20.5% reported a mother with asthma, 11.5% a father, 17.3% a sibling, and 12.8% a child. When comparing SCS use, no statistically significant differences were observed.

Regarding asthma severity, 37.3% of patients with mild asthma used SCS in the last 12 months, compared to 81.8% of patients with severe asthma. This difference was statistically significant (p<0.05). When analyzing the route of administration of SCS, 33.6% of patients received them via intramuscular or intravenous administration. For eosinophil levels, the median count among SCS users was 208 (range: 0-1630), whereas it was 114 (range: 0-920) among non-users, a difference that was also statistically significant (p<0.05).

Regarding asthma endotypes, 66.7%, 29.8%, and 83.3% of patients with low T2, allergic, and eosinophilic endotypes, respectively, used SCS. In terms of allergic comorbidities (rhinitis, conjunctivitis, food allergy, chronic urticaria, and atopic dermatitis), 100% (one patient) of those with all five diseases in addition to asthma used SCS. Among patients with two comorbidities, 43.8% (49 patients) used SCS. For passive smoking, 47.6% of patients who used SCS reported exposure, but the difference was not statistically significant.

Among obese patients, 61.8% had used SCS, compared with 44.9% of non-obese patients; however, this difference was not statistically significant. Although nasal polyposis was identified in only four patients, all of them had used SCS within the past 12 months (p<0.05). The principal findings are summarized in Tables 1-3, which detail the overall characteristics of the study population and compare patients who did and did not use SCS.

**Table 1:** Main characteristics and clinical findings among the study population (n = 152) Continuous variables were compared using the Student’s t-test or Mann–Whitney U test as appropriate. Categorical variables were analyzed with chi-square or Fisher’s exact test SD: standard deviation; NS: not significant

Variable	Category	Result	Observations/statistical findings	P-value	Test statistic (test used)
Age, years		Mean 42.9 (SD 1.75)	-	-	-
Sex	Women	113 (74.3%)	-	-	-
	Men	39 (25.7%)	-	-	-
Recruitment setting		79% from secondary-care hospitals	-	-	-
Asthma diagnosis	Spirometry	75.2%	-	-	-
	ICS response	24.8%	-	-	-
Systemic corticosteroid (SCS) use in the last 12 months		74 patients (48.7%)	-	-	-
SCS short courses in the last 12 months	≥1 course	17 (11.1%)	-	-	-
	≥3 courses	29 (19.0%)	-	-	-
Duration of asthma diagnosis	<1 year	7%	90% of <1 year patients used SCS vs. 48.4% of >5 years; difference NS	P>0.05	χ² = 2.95 (chi-square test)
	1–5 years	32.5%	-	P>0.05	-
	>5 years	60.5%	-	P>0.05	-
First-time consultation		10.2%	-	-	-
Family history of asthma	None	48.7%	No significant difference in SCS use	-	χ² = 1.27 (chi-square test)
	Mother	20.5%	-	P>0.05	-
	Father	11.5%	-	P>0.05	-
	Sibling	17.3%	-	P>0.05	-
	Child	12.8%	-	P>0.05	-
Asthma severity	Mild	37.3% used SCS	Statistically significant	P<0.05	χ² = 12.04 (chi-square test)
	Severe	81.8% used SCS	-	P<0.05	-
Route of SCS administration		Intramuscular/intravenous 33.6%	-	-	-
Eosinophil count, cells/μL	SCS users	Median 208 (0–1630)	Statistically significant	P<0.05	U = 2318 (Mann–Whitney U test)
Eosinophil count, cells/μL	Non-users	114 (0–920)	-	P<0.05	-
Asthma endotype	Low T2	66.7% used SCS	-	P>0.05	-
	Allergic	29.8% used SCS	-	P>0.05	-
	Eosinophilic	83.3% used SCS	-	P<0.05	-
Allergic comorbidities	All five (1 patient)	100% used SCS	-	-	-
	Two comorbidities (49 patients)	43.8% used SCS	-	-	-
Passive smoking exposure		47.6% of SCS users were exposed	Not significant	P>0.05	χ² = 0.51 (chi-square test)
Obesity	Obese	61.8% used SCS	Not significant	P>0.05	χ² = 2.01 (chi-square test)
	Non-obese	44.9% used SCS	-	P>0.05	-
Nasal polyposis		4 patients (100% used SCS)	Statistically significant	P = 0.03	Fisher’s exact test

**Table 2:** Demographic and clinical characteristics of patients who did not use SCS (n = 78) SCS: systemic corticosteroids; SD: standard deviation

Variable	Category	Values
Age, years, mean ± SD	—	48.5 ± 17.9
Sex, n (%)	Female	60 (76.9%)
	Male	18 (23.1%)
First-time consultation, n (%)	Yes	3 (3.8%)
	No	75 (96.2%)
Duration of asthma diagnosis, years, n (%)	>5	49 (62.8%)
	1–5	28 (35.9%)
	<1	1 (1.3%)
Asthma endotype, n (%)	Allergic	66 (84.6%)
	Eosinophilic	7 (9.0%)
	T2-low	5 (6.4%)
Allergic comorbidities, n (%)	Allergic rhinitis	69 (88.5%)
	Allergic conjunctivitis	4 (5.1%)
	Atopic dermatitis	2 (2.6%)
	Chronic urticaria	4 (5.1%)
	Food allergy	2 (2.6%)
Asthma severity, n (%)	Mild	37 (47.4%)
	Moderate	37 (47.4%)
	Severe	4 (5.1%)

**Table 3:** Demographic and clinical characteristics of patients who used SCS (n = 74) SCS: systemic corticosteroids; SD: standard deviation

Variable	Category	Values
Age, years, mean ± SD	—	37.0 ± 23.7
Sex, n (%)	Female	54 (73.0%)
	Male	20 (27.0%)
First-time consultation, n (%)	Yes	13 (17.6%)
	No	61 (82.4%)
Duration of asthma diagnosis, years, n (%)	>5	46 (62.2%)
	1–5	19 (25.7%)
	<1	9 (12.2%)
Asthma endotype, n (%)	Allergic	28 (37.8%)
	Eosinophilic	24 (32.4%)
	T2-low	22 (29.7%)
Allergic comorbidities, n (%)	Allergic rhinitis	64 (86.5%)
	Allergic conjunctivitis	7 (9.5%)
	Atopic dermatitis	8 (10.8%)
	Chronic urticaria	3 (4.1%)
	Food allergy	4 (5.4%)
Asthma severity, n (%)	Mild	24 (32.4%)
	Moderate	32 (43.2%)
	Severe	18 (24.3%)

## Discussion

In this study, we identified several characteristics of asthma patients that could guide the increased use of SCS. Understanding these characteristics is crucial for developing strategies to optimize the management of these patients and minimize long-term adverse events associated with their indiscriminate use. Our study found a significant relationship between SCS use and various clinical features in patients with asthma. One of the most notable findings is the higher use of SCS in patients with elevated eosinophil counts, reinforcing the role of eosinophilic inflammation as a key factor in asthma severity and management. This finding aligns with previous studies suggesting that SCS are frequently employed in patients with type 2 inflammation to prevent exacerbations and achieve better symptom control [15]. 

Additionally, SCS use was significantly higher in patients with severe asthma compared to those with mild or moderate asthma. This reflects the greater disease burden and challenges in achieving adequate symptom control in this population. However, trends in some countries indicate reduced use of these medications in severe asthma patients [16]. The reliance on SCS in these cases underscores the need for more targeted therapeutic alternatives, such as biologics, which have demonstrated the ability to reduce SCS requirements in severe asthma patients [17,18].

Interestingly, we found that patients with a recent asthma diagnosis (less than one year) also exhibited higher SCS use. This may reflect delays in diagnosis or initial disease presentations that are more severe or marked by exacerbations requiring acute treatment. Other studies have reported greater SCS use among patients with a longer duration since diagnosis, particularly in those with severe asthma and symptom onset at later ages [19]. One cohort even demonstrated that OCS use in asthma patients is more frequent in older women [20]. This finding highlights the importance of early diagnosis and appropriate treatment in the initial stages of the disease to prevent complications and severe exacerbations.

We did not find statistically significant differences in SCS use among patients with passive smoking, obesity, or varying numbers of allergic comorbidities; however, our study showed that obese patients tended to use SCS more frequently. Evidence already published supports the relationship between asthma and obesity [21]. Obesity itself is a factor that worsens asthma control. Regarding medication use for asthma management in obese patients, oral corticosteroids are commonly prescribed. A study conducted by Thompson et al. showed that oral steroid use is higher in obese asthma patients (OR: 1.86, 95% CI: 1.49-2.31; p<0.001, I² = 0%) compared to those with normal weight. Although not statistically significant, our data align with previous findings that obesity is linked to more frequent oral corticosteroid use and poorer asthma control [22]. 

Chronic rhinosinusitis with nasal polyps (CRSwNP) is among the most common comorbidities in asthma, especially in patients with eosinophilic inflammation [23]. The standard treatment for this condition includes local and systemic corticosteroids. A study by De Corso et al. demonstrated that short courses of oral steroids are commonly used for symptom exacerbations [24]. However, the use of these medications remains a subject of debate, and existing guidelines require further clarification. Although the number of CRSwNP cases in our study was limited, the consistent use of SCS among these patients indicates a potentially strong association that merits additional investigation.

This study has several limitations, including the retrospective review of some patient data, the use of convenience sampling, and the reliance on subjective measures for certain variables, such as family history of asthma. Additionally, because the sample consisted solely of patients treated in secondary- and tertiary-care institutions, selection bias may be present, which could limit the generalizability of the findings to populations managed in other healthcare settings. Despite these limitations, the results offer valuable local insights into corticosteroid use in real-life asthma management in our country.

## Conclusions

Increased SCS use was associated with eosinophilic asthma, nasal polyposis, recent asthma diagnosis, asthma severity, and obesity in our cohort. These findings highlight the urgent need for personalized asthma management, timely and accurate diagnosis, and a more thorough investigation of additional risk factors to help reduce excessive reliance on these medications and improve treatment outcomes for patients with asthma. Furthermore, strengthening steroid stewardship practices is essential to ensure that systemic corticosteroids are used judiciously and only when clinically indicated, particularly in patients with severe uncontrolled asthma. Our results highlight that systemic corticosteroid exposure remains frequent even among patients receiving regular inhaled therapy, suggesting persistent gaps in disease control and therapeutic optimization. Addressing these factors and implementing steroid-sparing interventions could help reduce both the clinical and systemic burden associated with asthma in real-world settings.
